# Towards Understanding Plant Calcium Signaling through Calmodulin-Like Proteins: A Biochemical and Structural Perspective

**DOI:** 10.3390/ijms19051331

**Published:** 2018-04-30

**Authors:** Valentina La Verde, Paola Dominici, Alessandra Astegno

**Affiliations:** Department of Biotechnology, University of Verona, Strada Le Grazie 15, 37134 Verona, Italy; valentina.laverde@univr.it (V.L.V.); paola.dominici@univr.it (P.D.)

**Keywords:** calcium-binding protein, calmodulin, plant calmodulin-like protein, *Arabidopsis*, EF-hand, conformational change, target-binding

## Abstract

Ca^2+^ ions play a key role in a wide variety of environmental responses and developmental processes in plants, and several protein families with Ca^2+^-binding domains have evolved to meet these needs, including calmodulin (CaM) and calmodulin-like proteins (CMLs). These proteins have no catalytic activity, but rather act as sensor relays that regulate downstream targets. While CaM is well-studied, CMLs remain poorly characterized at both the structural and functional levels, even if they are the largest class of Ca^2+^ sensors in plants. The major structural theme in CMLs consists of EF-hands, and variations in these domains are predicted to significantly contribute to the functional versatility of CMLs. Herein, we focus on recent advances in understanding the features of CMLs from biochemical and structural points of view. The analysis of the metal binding and structural properties of CMLs can provide valuable insight into how such a vast array of CML proteins can coexist, with no apparent functional redundancy, and how these proteins contribute to cellular signaling while maintaining properties that are distinct from CaM and other Ca^2+^ sensors. An overview of the principal techniques used to study the biochemical properties of these interesting Ca^2+^ sensors is also presented.

## 1. Introduction

As second messengers, Ca^2+^ ions have a fundamental role in a wide variety of environmental responses and developmental processes [1]. The process of signal perception and transduction through Ca^2+^ involves prompt changes in the levels of its intracellular free concentration that is used to coordinate a physiological response. In plants, continuous exposition to changing and potentially harsh conditions induces diverse spatial and temporal patterns of Ca^2+^ levels [2]. Referred to as “Ca^2+^ signatures”, these changes provide plants with information about external stimuli that are decoded using highly-specific protein sensors which trigger the appropriate physiological responses. Ca^2+^ sensors affect the activity of downstream effectors that synchronize changes in metabolism, gene expression, and turnover of proteins.

**Figure 1:**
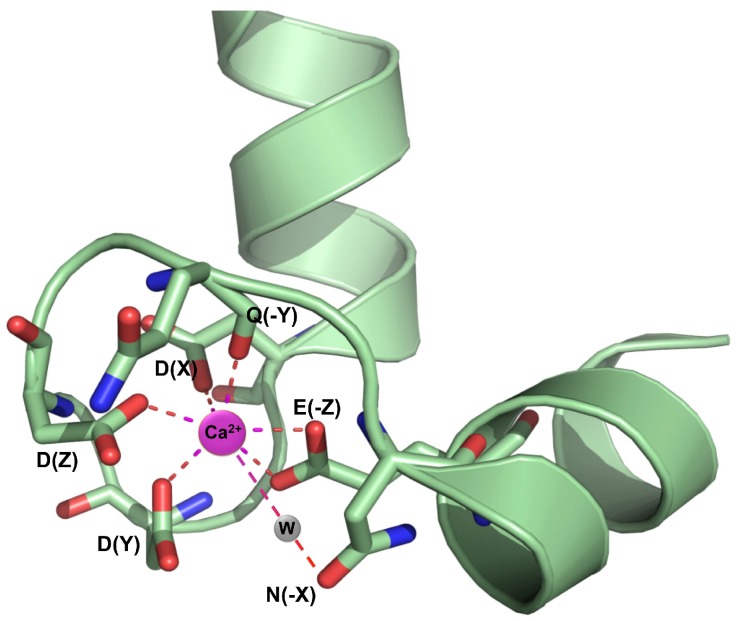
The canonical EF-hand. Ca^2+^ coordination in the Ca^2+^ binding loop-4 of *Arabidopsis* CaM7 (PDB CODE: 5A2H) [3]. W, water molecule. The image has been prepared using PYMOL (Schrödinger, LLC).

Most Ca^2+^ sensors contain a highly-conserved helix-loop-helix motif, called the EF-hand, which is composed of 29 amino acids; the central 12 residues form a loop structure that coordinates one Ca^2+^ ion (Figure 1).

The importance of Ca^2+^ sensors in growth and development of plants is highlighted by the diversity and large number of proteins identified to date with Ca^2+^ binding domains. There are three families of Ca^2+^ sensor proteins in plants: (i) calmodulin (CaM) and CaM-like (CML); (ii) calcineurin-B-like (CBL); and (iii) Ca^2+^-dependent protein kinases (CDPKs, called CPKs in *Arabidopsis*) [4,5,6]. Of these, only the latter represent true “responders” that carry out direct signal transduction using their own catalytic activity. CaMs/CMLs and CBLs appear to act as sensor relays that regulate downstream targets and are not endowed with catalytic activity. Nonetheless, CBLs can specifically interact with CBL-interacting protein kinases (CIPKs), which are a specialized group of serine/threonine protein kinases.

CaM is undoubtedly the best characterized Ca^2+^ sensor; it is highly conserved from an evolutionary standpoint and is present in all eukaryotic cells [7,8,9,10,11,12]. CaM is a small (149 amino acids) acidic protein. It has a flexible helical region in the center, which connects two globular domains. Each of these domains has two EF-hands that bind Ca^2+^ with positive cooperativity. In addition to multiple CaM isoforms, plant genomes encode a remarkable number of CMLs whose primary sequences have ≥16% overall identity with the canonical CaM sequence (e.g., CaM2 from *Arabidopsis*); functional motifs other than EF-hands are notably absent [9,13]. In *Arabidopsis*, seven genes are present that code for four CaM isoforms (CaM1/4, CaM2/3/5, CaM6 and CaM7) as well as 50 genes that code for CML proteins [9,13]. The successful completion of several plant genome sequencing projects has allowed for identification of many genes that are predicted to encode CML isoforms in various plant species (e.g., 32 in *Oryza sativa* [14], 52 in tomato [15], 36 in woodland strawberry [16], 19 in *Lotus japonicus* [17], and 79 in Chinese cabbage [18]), revealing the high level of diversity of Ca^2+^ sensors in the green lineage [4].

The functions of *Arabidopsis* CMLs in development and response to both biotic and abiotic stimuli have been summarized in several recent reviews, and convincing evidence has been provided that these proteins are not likely to have redundant functions, but rather play central and highly specific roles in coordinating environmental responses of plants. In addition, many CMLs are now known to recognize a specific target [2,19,20,21,22,23].

Empirical data on the affinity of Ca^2+^ to CMLs and the Ca^2+^-induced structural rearrangements are just beginning to emerge. Importantly, only one 3D atomic structure of a CML is present in the Protein Data Bank (PDB), i.e., the Ca^2+^-loaded form of the N-terminal domain of CML34 from *Arabidopsis thaliana* (PDB code 1TIZ) obtained by protein nuclear magnetic resonance (NMR) spectroscopy [24], while X-ray crystallographic structures of the apo- and Ca^2+^-bound forms of CMLs are still missing. Nevertheless, it has been possible to obtain useful data about the dynamic properties of CMLs using combined biochemical approaches, which include isothermal titration calorimetry (ITC), NMR, circular dichroism (CD), and fluorescence spectroscopy. The emerging scenario is that CMLs are a highly diverse family of proteins that act generally, but not always, as Ca^2+^ sensors, and provide a wide variety of physiological responses to Ca^2+^.

The present review discusses and summarizes the current knowledge on plant CMLs, from both biochemical and structural perspectives, and provides an overview of the principal techniques used to study these Ca^2+^ sensors. The majority of the examples given within come from members of the *Arabidopsis* CML family since there is more biochemical, structural, and functional information for this family than for any other plant species.

## 2. The EF-Hand: Variations on a Theme

### 2.1. Architecture of EF-Hands

As a metal ion, Ca^2+^ has the ability to provide interactions that are dominated by ionic forces [25]. Accordingly, the EF-hands, and in particular the loops, are abundant in negative charged glutamate and aspartate residues. The EF-hand loop provides seven ligands that can bind Ca^2+^ with pentagonal bipyramid geometry. In particular, the Ca^2+^ ion is coordinated in the canonical EF-hand to carboxylate oxygens from residues 1 (+X), 3 (+Y), 5 (+Z) and 12 (-Z), carbonyl oxygen from residue 7 (-Y) and bridged water at position 9 (-X). A glycine residue at position 6 is highly conserved, allowing the loop to encompass the Ca^2+^ ion, which is a critical feature for high affinity binding [26] (Figure 1). The EF-hand is a structural and functional unit as well as a unit of evolution. Accordingly, a recent classification of subfamilies of EF-hand proteins has provided evidence that the majority of EF-hand proteins probably evolved from one ancestral EF-lobe (a pair of adjacent EF-hands) [27].

Sequence alignment of plant CaM proteins has documented a conserved pattern DxDx[DN] in the EF-hand binding loop, in which aspartate and asparagine are most commonly present, indicating that the short branch length of these residues is optimal for Ca^2+^ binding at positions 1, 3, and 5. Residue 12(-Z) is glutamate in most Ca^2+^-binding EF-hand motifs, thereby providing bidentate chelation. Though non-coordinating residues, glycines at position 4 and 6 determine in large part the flexibility in the Ca^2+^ site. Hydrophobic amino acids (I, V, or L) are predominantly present at position 8 (Figure 1).

**Figure 2:**
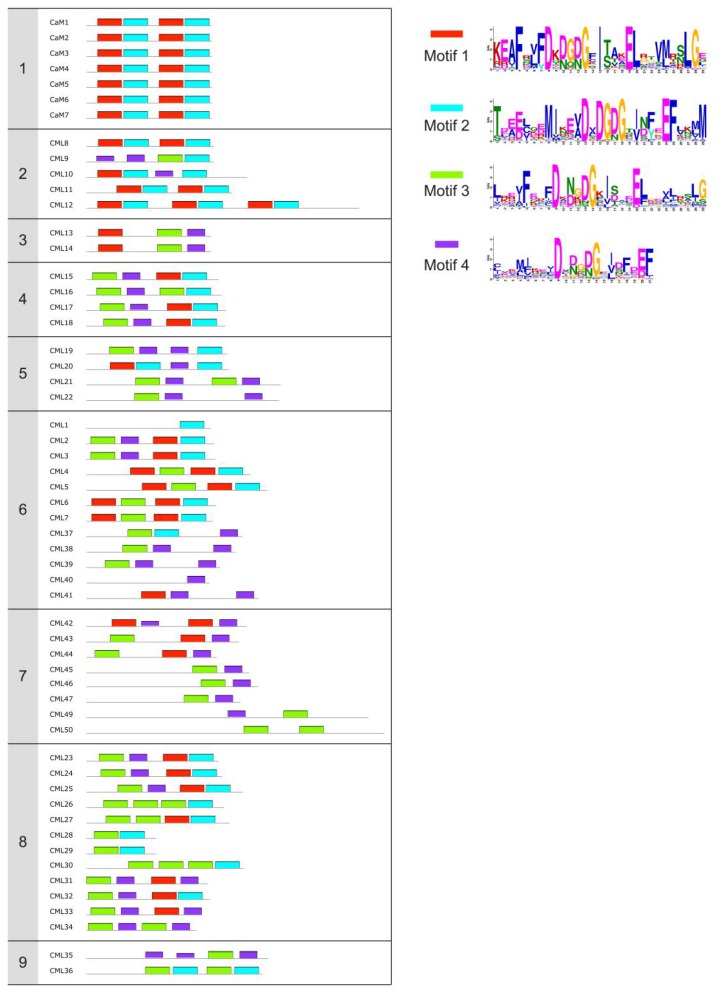
EF-hand motifs composition of *Arabidopsis* CaM and CML proteins. Conserved motifs were identified using the Multiple Em for Motif Elicitation (MEME) suite (http://meme-suite.org/tools/meme) [28] with standard searching parameters, a maximum of four motifs and an optimum motif width between six and 29 amino acids. Each color represents a specific motif for which the corresponding sequence LOGO is shown on the right side of the panel. The seven CaM and 50 CML proteins are clustered into nine groups according to [13]. Not all motifs found are actually functional and differences exist between the predicted motifs by MEME and PROSITE-ProRule (Table 1).

Plant CMLs have a structural similarity to CaM and are also predicted to possess EF-hands, with no additional functional domains. While CaM typically contains four conserved EF-hand motifs, CMLs generally have one to six [4,13,14,15,16,17]. As an example, CaMs from *Arabidopsis* have 149 amino acids and four EF-hands; CMLs range from 80 to 330 amino acids in length and 16 of 50 CMLs have a number of EF-hands that is different from four (Figure 2). Sequence analysis of CMLs from several plant species has highlighted that both the composition and organization of functional EF-hands in CMLs have significant variations [4,13,14,15,16,17]. A variability within and between loops can be found and different residue positions distinguish each Ca^2+^-binding site. Due to the lack of key coordinating residues and deletions in the EF-binding loop motifs, several domains are likely non-functional and thus not even recognized by bioinformatics tools searching for motifs. In many cases, residues with a negative charge (needed to bind Ca^2+^) are substituted by positively charged polar residues (e.g., the presence of a lysine residue at positions 3, 6 and 12 in the second EF-hand of CML17, CML22, CML25, and CML33) that can perturb the network of interactions needed for efficient binding. At position 12, the substitution of glutamate with aspartic acid residue is very frequent among CMLs (Figure 2). This is of interest as aspartate is known to shift the binding selectivity from Ca^2+^ towards Mg^2+^ ions [26]. Furthermore, the binding of Ca^2+^ can be altered by mutation of loop residues at non-critical positions (Figure 2) as well as by the three-dimensional arrangement of the two helices, which leads to the abandonment of the pentagonal bi-pyramidal coordination scheme and the acquisition of non-canonical binding geometry. Accordingly, it could be hypothesized that non-identical loops may determine functional flexibility in the binding of Ca^2+^ due to their different biophysical properties. There is thus a need for in-depth investigation of the contribution of each EF-hand loop to the functions of CMLs considering the crucial residues that distinguish the loops from one another.

### 2.2. The Affinity of EF-Hands for Ca^2+^

Only a few CMLs have been incontrovertibly demonstrated to function as Ca^2+^ sensors. ITC and NMR are ideal techniques to better understand the intricacies of Ca^2+^-binding proteins. In fact, while ITC allows for determination of the thermodynamics for multiple metal-binding sites, NMR can unequivocally identify the stoichiometry of binding. Indeed, the appearance of downfield-shifted ^1^H resonances at >10 ppm in ^1^H-^15^N HSQC spectra is characteristic of Ca^2+^-loading of EF-hand-containing proteins. Such signals are typical of the backbone amide groups of the conserved glycine at position 6 of the EF-hand binding loop (G_6_) that helps form the large hydrogen-bonding network upon Ca^2+^ binding [29]. Thus, G_6_ functions as an indicator of the Ca^2+^-bound state in an EF-hand. Using ITC and NMR data, the existence of three functional Ca^2+^ binding sites has been demonstrated for *Arabidopsis* CML42 [30] and CML43 [31], and four for CML19 [32] and CML36 [33] (Table 1). In the case of CML14, the combination of ITC and NMR techniques has allowed for the demonstration that only one EF-hand can sense Ca^2+^ ions, despite the presence of three EF-hand motifs [34]. Moreover, ITC analysis demonstrated that CML15 and CML16 contain only two and three functional Ca^2+^-binding sites, respectively, out of the four predicted EF-hands [35]. Therefore, caution should be used in functional predictions based only on sequence analysis (Table 1). In the absence of experimental 3D structures, homology models can be exploited as an alternative to support the presence of functional Ca^2+^-binding sites, as has been done for CML14 [34], CML15, and CML16 [35].

ITC analysis has underscored that *Arabidopsis* CMLs have a wide range of affinity for Ca^2+^ (nM-µM range) [30,31,33,35]. Thus, the Ca^2+^ signaling system might be endowed with greater flexibility as a consequence of the different Ca^2+^ binding affinities of the various isoforms of CML. In this regard, a range of Ca^2+^ sensors for several Ca^2+^ signatures is likely to represent a crucial aspect for Ca^2+^ signal transduction. In addition, it would further appear that most CMLs have an apparent affinity for Ca^2+^ which differs substantially from CaM itself, leading to the possibility that they might be activated differently during Ca^2+^ spikes. The different binding affinities of *Arabidopsis* CML36 and CaM for Ca^2+^ ions would be critical in fine-tuning each isoform to specifically stimulate the activity of the common target, the Ca^2+^-dependent ATPase isoform 8 (ACA8), in different conditions [33]. A tobacco CML (rgs-CaM), which was reported to possess an associated RNA silencing suppressor activity [36], has a Ca^2+^-affinity which is not in the range of canonical CaMs [37]. In particular, ITC analysis indicated that the protein possesses two Ca^2+^-binding sites with moderate Ca^2+^ affinity and a third one with very low Ca^2+^ affinity (*K*_d_ ~ 10 mM). This low/modest affinity has been attributed to hydrophobic amino acid substitutions within the EF-hands, especially in EF-hands 1 and 4 [37]. However, due to the high *K*_d_, it is questionable whether rgs-CaM can work as a true Ca^2+^ sensor in vivo, even if its affinity for Ca^2+^ could increase in the presence of targets. Indeed, association of CaM with its targets is known to stabilize its Ca^2+^-bound conformation, increasing the affinity for Ca^2+^ [10,26,38]. 

### 2.3. The Role of Mg^2+^

It is worth noting that, in addition to Ca^2+^, Mg^2+^ is another physiologically important ion for plants. In plant cells, the free cytosolic concentration of Ca^2+^ and Mg^2+^ in the resting state has been reported to be about 100 nM and 0.5–2 mM, respectively [39,40]. This renders the possibility that the competitive and/or allosteric effects of Mg^2+^ are relevant. Indeed, since Ca^2+^ and Mg^2+^ have similar properties, Ca^2+^-binding proteins must be able to discriminate between the two cations against a 10^2^–10^4^ -fold excess of Mg^2+^. However, it has been shown that Mg^2+^ binding to EF-hands is important physiologically, and in reality more than one role has been hypothesized for the binding of Mg^2+^ [26,40]. These include providing greater structural stability to a molten globule apo-protein, as well as a potential role in modulating the affinity of EF-hands for Ca^2+^. Considering this possibility, binding of Mg^2+^ might play a functional role by shifting an activation curve to higher concentrations of Ca^2+^ while inactivating other enzymes at resting levels of Ca^2+^. In some Ca^2+^-binding proteins, it is known that Mg^2+^ binding has a function that is distinct from Ca^2+^ [41].

*Arabidopsis* CMLs show heterogeneous behavior towards Mg^2+^ ion binding: in CML19 [32] none of the four EF-hands can bind Mg^2+^, while in CML14 [34], CML15, and CML16 [35] the weaker affinity for Ca^2+^ in the presence of Mg^2+^ indicates that this cation can compete directly for Ca^2+^ binding [26,42,43,44,45], thereby reducing the affinity for Ca^2+^ by 5-10 fold. For CML16 [35], Mg^2+^ binding seems to impede the binding of Ca^2+^ to at least one EF-hand. Mg^2+^ also affects the affinity for Ca^2+^ in CML36 which possesses two Ca^2+^/Mg^2+^ mixed sites with high affinity and two Ca^2+^-specific sites with low affinity [33]. The observed binding constants of Ca^2+^/Mg^2+^ mixed sites for Mg^2+^ and Ca^2+^ are suggestive that both these EF-hands are normally occupied by a divalent cation during the resting state. This ensures that CML36 is in a folded ion-bound structure at all concentrations of Ca^2+^. After a stimulus-induced Ca^2+^ increase, Mg^2+^ is displaced and the dominant state of the protein becomes Ca^2+^-bound [33]. This finding demonstrates that Mg^2+^ binding does not preclude the ability of CMLs to functionally respond to Ca^2+^.

It is worth pointing out that, in some cases [30,31], the affinity for Ca^2+^ has been measured exclusively in the presence of Mg^2+^ to mimic physiological conditions. While the approach appears to be theoretically valid, the study of Ca^2+^ binding in the absence and presence of Mg^2+^ may be crucial for understanding protein functionality. Indeed, performing NMR and ITC titrations of apo-CMLs with Ca^2+^ or Mg^2+^, as well as titration of Ca^2+^ in CMLs saturated with Mg^2+^, will provide crucial information on the possible competition between the two ions and on the influence of Mg^2+^ binding on Ca^2+^ affinities.

The biochemical data on CMLs, albeit limited, give added credit to the hypothesis that the heterogeneity in the organization and composition of the EF-hands in CMLs is at the basis of their functional diversity, either by allowing activation at specific Ca^2+^ spikes due to a specific stimulus or through selective interaction with precise targets. Preserving multiple CML proteins may be essential in complex organisms to guarantee that the many Ca^2+^-dependent processes occur with the appropriate spatial-temporal resolution. This hypothesis may also explain the presence of 12 highly homologous (>70% identity) pairs of proteins [13] that could be derived from relatively recent duplication events and successive diversification (e.g., CML13 and CML14, CML35 and CML36, CML15 and CML16, and CML17 and CML18). Of course, the presence of nearly-identical isoform pairs may have other explanations, including redundancy, which would not necessarily point to a specific role of CMLs. Unfortunately, there is not yet sufficient information about the functional properties of these pairs, although recent biochemical characterization of the two closely-related paralogs CML15 and CML16 [35] appears to demonstrate that subtle differences in the composition of the EF-hands can be associated with specific differences in the response to Ca^2+^.

It is also interesting to note that the structure of the *CML* genes, including their intron/exon organization, has significant differences from that of *CAMs*. Indeed, the majority of *CML* genes are intron-less, while those of *CAMs* are intron rich [15,16,18,92]. There is not yet clear information, from an evolutionary perspective, about the presence of introns in eukaryotic genes. However, in accordance with the introns-late hypothesis [93] and recent literature [92], CMLs may have evolved earlier than CaMs and diversified later [92]. Therefore, it is possible that evolution led to a specific role for CMLs in plants.

## 3. Structural Consequences of Ca^2+^ Binding and Conformational Changes

Conventionally, the role of Ca^2+^ binding has been looked at in terms of signal transduction, focusing on Ca^2+^-induced conformational changes and what effects these may have on interactions with specific targets. This allows distinguishing Ca^2+^ sensors from what is generally referred to as “Ca^2+^ buffers” (exemplified by human calbindin D9K and parvalbumin [94]), which have high affinity for Ca^2+^ and undergo minimal conformational changes upon binding of Ca^2+^. These proteins have been presumed to chelate Ca^2+^, which is potentially toxic for the cell.

A conformational change in CaM involves the transition from a “closed” apo-state to an “open” holo-state that is portrayed by an enlarged interhelical angle of the EF-hand, leading to alterations in the protein surface from a predominantly hydrophilic to a more hydrophobic state when Ca^2+^ is bound. This is largely due to exposition of a hydrophobic region that is rich in Met residues (e.g., 6% in *Arabidopsis* CaMs) which were previously embedded within the protein. Through changing exposed surfaces, it is interesting that CaM regulates more than 300 proteins, including kinases, phosphatases, receptors, pumps, and channels [95,96,97,98,99,100]. Such Ca^2+^-induced changes in surface hydrophobicity can be utilized for purification of many recombinant Ca^2+^ proteins by using hydrophobic interaction chromatography (HIC). The Ca^2+^-dependent binding to phenyl-sepharose can be considered as a first step in studying a Ca^2+^ sensor protein. Along with this, the finding that CaM has increased mobility in electrophoresis if Ca^2+^ is present is, in fact, a defining property that can be used as another simple approach when investigating putative Ca^2+^ sensors [101]. Several CMLs were found to display Ca^2+^-dependent electrophoretic mobility shifts via SDS-PAGE (Table 1) [30,31,32,33,37,46,48,64,70,73,77,80,88,102], although such shifts are often less dramatic than those seen with CaM.

Significant information on the structural rearrangements that CMLs undergo upon addition of metals can be obtained by 2D ^1^H^15^N HSQC NMR spectra of uniformly ^15^N-labeled recombinant CMLs, even in proteins for which conformational changes are difficult to detect in mobility shift assays or on phenyl-sepharose. In this regard, the ^1^H^15^N HSQC spectra of apo-CML19 [32], CML42 [30], and CML43 [31], while showing characteristics of well-folded proteins, change intensely upon the addition of Ca^2+^, with the appearance of several well-dispersed peaks and numerous peaks that experience chemical shift variations. This implies that these proteins undergo a conformational rearrangement before acquiring a well-ordered structure. Of interest, the NMR spectra of apo-CML36 is defined by fewer peaks than would be expected, considerable line broadening, and low dispersion of chemical shift, thereby suggesting that the apo-protein has a loosely folded conformation, probably similar to a molten globule [33]. Cation binding (both Mg^2+^ and Ca^2+^) to Ca^2+^/Mg^2+^ mixed sites appear to guide the change from a molten globule apo-structure to a stable holo-protein. However, when examining the position of peaks in the forms complexed with Mg^2+^ and Ca^2+^, it is clear that the conformational changes in CML36 induced by binding of Ca^2+^ are distinct from those induced by Mg^2+^, in agreement with its hypothesized function as a Ca^2+^ sensor [33].

Crucial structural information on CML proteins and their Ca^2+^ binding ability can also be obtained by CD spectroscopy in the far-UV region. Multiple lines of evidence have indicated that CMLs contain substantial α-helical structure as for CaM. Nevertheless, in contrast to CaM for which a distinct increase in ellipticity has been observed in the presence of Ca^2+^, the behavior of CMLs is somewhat more variable. CD data for *Arabidopsis* CML15 [35] is reminiscent of CML43 [31] and CML42 [30], since the binding of Ca^2+^ has almost no impact on secondary structure. A modest effect on the CD spectrum upon addition of Ca^2+^ was also observed in tobacco CML (rgs-CaM) and soybean CML27 [37,103]. However, in *Arabidopsis* CML16 [35], CML37 [82], CML39 [85], and CML36 [33] the binding of Ca^2+^ increases the overall helical content. Rice CMLs (OsCMLs) also have heterogeneous behavior in terms of structural changes in CD spectroscopy with some members of the family displaying small changes (e.g., OsCML1, OsCML3, and OsCML9) and others showing large increases in molar ellipticity (OsCML4, OsCML5, OsCML8, OsCML11, and OsCML13) following binding of Ca^2+^ [102]. It is still unclear what role such apparently small structural alterations have on the function of different CMLs. Notwithstanding, this demonstrates that this sizable family of Ca^2+^ sensors is much more complex than originally believed. For CaM, variations in the response to Ca^2+^ binding mainly involve helix reorientation, and not merely a change in α-helical content [104], uncovering hydrophobic portions that are likely needed for association with various targets [26,105]. In particular, the existence of a large proportion of Met residues gives CaM the conformational plasticity to fine-tune itself to a variety of targets [106,107,108]. The mean percentage of Met residues detected in *Arabidopsis* CMLs (4.2%) does not differ substantially from that in CaM, suggesting that CMLs could share a conserved and analogous mechanism of action with CaM. Nonetheless, it should be noted that the Met content in *Arabidopsis* CMLs ranges from 0.9% to 8.6% and that the amount of exposed hydrophobic surfaces, in the apo- and holo-forms of CMLs, do indeed vary when considering the different family members, as demonstrated by studies with the fluorescent probe anilino-8-naphthalene sulfonate (ANS). CML36 (2.4% Met) is similar to CML15 (4.5%) and CML16 (5.0%) in that they show a significant degree of hydrophobic exposure even when Ca^2+^ is not present and only a relatively small increase in hydrophobicity is observed when Ca^2+^ is bound [33,35]. On the other hand, CML19, CML37, CML42, and CML43, which possess 6.6%, 4.3%, 2.6%, and 2.2% Met, respectively, are more similar to CaM, as they display a low level of exposure of hydrophobic residues in the apo- form that augments substantially when bound to Ca^2+^ [30,31,32,82]. Remarkably, CML14 binds only one Ca^2+^ atom without changes in exposed hydrophobicity, and therefore it does not behave like a classical Ca^2+^ sensor [34]. On the other hand, the presence of a single low affinity Ca^2+^ binding site is unlikely to be compatible with a buffer function. The behavior of CML14 could point out a role of Ca^2+^ for target binding of CML14 that differs from the classical switch-like role with exposure of the interfacial hydrophobic regions. Only the identification of the interaction partners of CML14 will elucidate its molecular mechanisms of action. Notably, rice CMLs also exhibit a broad spectrum of hydrophobic characteristics as measured by ANS fluorescence [102]. This structural multiplicity in CMLs is in line with the likelihood that they have divergent yet overlapping roles as Ca^2+^ sensors and further implies that the binding of a target to a CML might be based on a recognition mechanism that is more specific than just generalized exposure of hydrophobic residues.

## 4. Interaction of CMLs with Targets

The recognition of targets for CaM/CML and better appreciation of the impact of CaM/CML-binding on biological processes are primary goals in untangling the broader role of CaMs/CMLs. Up to now, several CML targets have been identified by protein microarray analysis, in addition to genetic and in vivo studies. The protein targets identified to date include transcription factors, protein kinases, metabolic enzymes, and transport proteins [2,51,60,64,109,110,111]. These investigations have assigned relatively specific physiological roles to several CMLs (Table 1). On the one hand, the identification of specific targets for some CMLs (e.g., CML8, CML18, CML19, and CML20 [52,53,66,67,111]) (Table 1) suggests that they can have diverse roles in both plant development and response to stress, different from CaM, which has broad target specificity. This brings the question of which variations in structural features, and especially of the binding pocket, might define the target specificity in CMLs. On the other hand, some CMLs (CML9 [52,58,59,62], CML24 [73,74,76], CML37 [81,82], CML38 [84], and CML42 [30]) have been shown to act at crucial points in various signaling pathways, perhaps by helping plants to handle diverse environmental challenges. Therefore, at least some CMLs might behave as gateways, being able to assimilate signals from biotic and abiotic stimuli, driving signaling pathways towards a desired response. Importantly, protein microarray analyses [110] and detailed analyses of specific CMLs [33,56] suggest the existence of overlap between CaM and CMLs targets. The choice of the most appropriate signaling pathway involving CaM or CML to provide a specific downstream response following a stimulus may depend on several factors such as the spatio-temporal expression of the protein, characteristics of Ca^2+^ signals, and molecular properties of the two EF-hand proteins (e.g., affinity for ions, conformational response to ion binding, and post-translational modifications). Therefore, CMLs might be able to carry out interactions that are common among the different members of the protein family, in addition to interactions that are specific to individual members. Moreover, the interaction of CML proteins with other CML family members has been documented, which could be significant in terms of Ca^2+^ signaling events [110].

CaM-binding domains (CaMBDs) normally share similar secondary structures consisting of short (12–30 amino acids) sequences of amino acids with a tendency to form α-helices [112]. These structures can interact with the hydrophobic regions in CaM that are uncovered following Ca^2+^ binding. In addition, electrostatic interactions between CaM and a target CaM binding domain can lead to stabilization of a CaM-target complex [26,113]. The ability of CaM to engage diverse targets arises both from the plasticity of the linker region connecting its globular domains, which allows CaM to wrap around the target, and from the multiple conformations adoptable by the exposed hydrophobic cleft thanks to the flexibility of Met side chains [26,98,113,114]. Moreover, CaM also interacts with proteins even in the absence of Ca^2+^, which reveals its versatility in terms of signaling [113].

Multiple sequence alignment between CaMs and CMLs highlights two major differences that may be associated with an important impact on structure and target interactions. First, CMLs are widely variant in length compared to CaM, and have an N- or C-terminal extension in which signal sequences are not always readily found (Figure 2). These extensions may bring about the existence of a complex structure that is different from CaM, and thus CMLs might not be able to wrap around their target but rather bind with a different conformation. Moreover, the possible presence of a linker region with different length and low sequence homology between CaMs and CMLs could represent a significant difference in defining the flexibility of CML proteins, and thus their ability to interact with targets [115]. As one example, a tobacco CML was reported to interact with its targets via electrostatic interactions [37,116], in contrast with the canonical CaM binding mechanism which is mainly hydrophobic.

In the interaction with target, the presence of secondary modifications is crucial since these can play particularly important roles in protein function and regulation. Different CMLs (e.g., CML21 from *Arabidopsis*, CML5 and CML11 from tomato [15], and CML14 and CML18 from *Lotus japonicus* [17]) harbor a predicted canonical consensus N-myristoylation motif. Overall, the existence of co- or post-translational N-myristoylation is suggestive that potential targeting of CaMs/CMLs to membranes might be an important aspect of their function, especially in plant defense responses. When combined with N-myristoylation, the existence of several phosphorylation sites in plant CMLs [4,13,14,15,16,17] could potentially give rise to a large number of species with distinct properties. Moreover, many CMLs (e.g., CML23, CML24, CML25, CML26, CML27, CML33, CML35, CML36, and CML37 from *Arabidopsis*) have pairs of cysteines that can form disulfide bonds that affect the structural properties of the protein, e.g., allowing dimerization, and target binding. Since the EF-hand is normally present in pairs, dimerization could explain the existence of CMLs with odd numbers of functional EF-hands.

Thus, greater knowledge of CML-target complexes is needed, and understanding the specific roles of Ca^2+^ sensors will require the study of their regulation. A major challenge will be to evaluate the structural properties and functional aspects of target binding. Certainly, the 3D structures of the apo- and holo-forms and of the complex with their target will be needed to categorically address these issues, to compare the recognition mode, and get deeper insight into the structural diversity of CML-binding to their target regions. Besides X-ray crystallography and NMR spectroscopy, cryo-electron microscopy (cryo-EM), which emerged as a remarkably successful technique for protein structure determination in the latest years, can also provide useful information on CML-target complexes (provided that CML-target complexes of sufficient size are studied). Notwithstanding, an interesting approach to study the interactions between CaM/CML-target is by identification of the CML-binding region in the target and synthesizing the corresponding peptide. Different biophysical techniques, including fluorescence, NMR and CD spectroscopy as well as ITC, SEC and native-PAGE, in fact, can be used to perform thorough structural and energetic characterization of the CML-peptide interaction and its Ca^2+^ dependence. Such approaches have been applied for many Ca^2+^ sensors, and not only in plants [10,113,117,118,119,120,121]. However, among *Arabidopsis* CML members the only CML-target complexes for which a detailed biochemical description has been achieved are CML19-RAD4 [32] and CML36-ACA8 [33].

A first simple analysis is monitoring the complex formation between CML and the target peptide via native PAGE. Indeed, upon incubation of the protein with increasing molar ratio of the peptide the appearance of a new band with a lower mobility than that of free CML is a clear indication that a protein-peptide complex has been formed [10,32,33]. For example, non-denaturating gel band shift electrophoresis directly demonstrated that the peptide representing the CML19-binding site on RAD4 (RAD4p) forms 1:1 complex with Ca^2+^-saturated CML19 [32]. Moreover, native PAGE analysis has confirmed the ability of CML36 to interact with the N-terminus of ACA8 [33].

Next, Trp fluorescence spectroscopy can give crucial information on the stoichiometry and binding strength, as well as the mode of binding. Trp is often considered as an intrinsic fluorescent probe to follow conformational changes. Several binding regions in CaM/CML proteins contain a lone Trp residue [98], whereas CaM and many CMLs have no Trp. The formation of a CML-target complex is accompanied by a significant blue shift and increase in intensity of Trp emission fluorescence. These changes are indicative of an interaction between protein and target that gives rise to insertion of Trp from a polar to a non-polar environment. The addition of the Ca^2+^-saturated CML19 to RAD4p caused a significant increase in the fluorescence intensity of the peptide and a blue shift of maximum emission wavelength from 353 to 333 nm, indicating that the only Trp in the peptide entered a more hydrophobic environment and confirming that RAD4p interacts with Ca^2+^/CML19 [32].

Far-UV CD spectroscopy can complement Trp fluorescence as a basic tool to study the interaction of CML with peptide, since many CaM/CML-binding peptides are placed into an amphipathic helix after binding Ca^2+^ [11,122,123,124]. Normally, the peptide alone in the presence of Ca^2+^ has an unordered structure. Addition of the peptide to the protein usually leads to an increase in the dichroic signal. A smaller rise in ellipticity signal could be associated to conformational changes of the Ca^2+^ sensor itself, but the major contribution usually comes from the peptide, changing from random coil to α-helical following interaction with the protein [11,110,125]. This conformational change has been observed for RAD4p upon incubation with Ca^2+^-CML19, indicating that RAD4p might be induced to adopt α-helical structure [32]. Following addition of the peptide to Ca^2+^-saturated CML19, the NMR spectrum of the protein also underwent considerable changes with some peaks undergoing chemical shifts, and new peaks appearing, thereby confirming that the interaction between CML19 and the peptide leads to a unique, stable structure [32].

Finally, it should be mentioned that the thermodynamic parameters of peptide binding to CMLs can be determined using ITC, which also gives crucial information about the dominant forces in the association of the peptide with the specific CML (electrostatic versus hydrophobic interaction). However, up to now, such an approach has never been used to study the energetics of CML-target interactions.

Clearly, there is a lack of biochemical and biophysical characterization on the binding of CMLs to their targets (and/or peptides). Further studies of the interaction of CMLs with several natural peptide targets, as well as CaM-specific targets, in addition to solving the structure of Ca^2+^-CML complexes, will undoubtedly provide more insights into the molecular basis of the activity of CMLs.

## 5. Conclusions

While not exhaustive, we have attempted to summarize the recent advances in our understanding of the features of CMLs from biochemical and structural points of view (Table 1). To learn more about the functional role of CMLs, additional information on physiological features must be supplemented with detailed analysis of both the metal (Ca^2+^ and Mg^2+^) binding and structural properties. One of the major challenges will be obtaining 3D structures of the holo- and apo-CMLs in isolation and in complex with targets. Moreover, there is a need to expand the knowledge about the roles of post-translational modifications on CMLs which are strongly related to the biological activity of proteins. Multiple channels of evidence have indicated that CMLs have the biochemical properties of Ca^2+^ sensors. Globally, biochemical and structural analysis of CMLs will provide insight into how such a vast array of CMLs proteins can coexist, without apparent redundancy, and how they make a distinct contribution to cellular signaling while being different from CaM and other Ca^2+^ sensors.

## Figures and Tables

**Table 1:** Summary of available structural and functional information on *Arabidopsis* CMLs.

Name ^1^	Accession number	EF-hands ^2^	Experimental Ca^2+^-binding sites ^3^	Biochemical and structural characterization ^4^	Identified target	Putative role	Refs
CML1	At3g59450	1	?	?	?	?	
CML2	At4g12860	4	?	?	?	?	
CML3	At3g07490	4	?	Gel shift, HIC	AtDEG15	?	[46,47]
CML4	At3g59440	4	?	Gel shift, HIC	?	?	[48]
CML5	At2g43290	4	?	Gel shift, HIC	?	?	[48]
CML6	At4g03290	4	?	?	?	?	
CML7	At1g05990	4	?	?	?	Development (Root hair elongation)	[49,50]
CML8	At4g14640	4	?	HIC, radioactive Ca^2+^-binding assay	BRI1, ZAR1, IQD1, PEN3	Plant immunity (Positive regulation)	[51,52,53,54,55,56,57]
CML9	At3g51920	4	?	?	PPR2, IQD1, PEN3, ILK1	Signaling hub ^5^	[52,55,56,58,59,60,61,62,63]
CML10	At2g41090	4	?	Gel shift	PM-MUTASE	Abiotic stress (Oxidative stress)	[64]
CML11	At3g22930	4	?	?	?	?	
CML12	At2g41100	6	?	?	PINOID, PEN3	Development; Plant immunity	[56,65]
CML13	At1g12310	3	?	?	?	?	
CML14	At1g62820	3	1	NMR, ITC, DSC, Gel shift, ANS, SEC, LP, MM	?	?	[34]
CML15	At1g18530	4	2	Gel Shift, CD, ANS, ITC, HIC, MM	?	?	[35]
CML16	At3g25600	4	3	Gel Shift, CD, ANS, ITC, HIC, MM	?	?	[35]
CML17	At1g32250	4	?	?	?	?	
CML18	At3g03000	4	?	?	NHX1, CBP60C	Abiotic stress (Salt)	[66]
CML19	At4g37010	4	4	NMR, ITC, Gel shift, ANS, SEC, CD, LP	RAD4, SAC3b, DSS1	Abiotic stress (UV-damage)	[32,67,68,69]
CML20	At3g50360	4	?	Gel shift	TON1, SAC3, UCH	Abiotic stress (Drought Stress)	[69,70,71]
CML21	At4g26470	4	?	?	?	?	
CML22	At3g24110	4	?	?	?	?	
CML23	At1g66400	4	?	?	?	Development (Flowering)	[72]
CML24	At5g37770	4	?	Gel shift, HIC	ATG4b	Signaling hub^5^	[72,73,74,75,76]
CML25	At1g24620	4	?	Gel shift, HIC	?	Development (Root, Pollen tube)	[77]
CML26	At1g73630	4	?	?	?	?	
CML27	At1g18210	4	?	?	?	?	
CML28	At3g03430	2	?	?	?	?	
CML29	At5g17480	2	?	?	?	?	
CML30	At2g15680	4	?	Gel shift, HIC	?	?	[46]
CML31	At2g36180	4	?	?	?	?	
CML32	At5g17470	4	?	?	?	?	
CML33	At3g03400	4	?	?	?	?	
CML34	At3g03410	4	?	NMR	?	?	[24]
CML35	At2g41410	4	?	?	TTL3	?	[78]
CML36	At3g10190	4	4	NMR, ITC, DSC, Gel shift, ANS, SEC, LP, CD	ACA8, CERK1	?	[33,79]
CML37	At5g42380	4	?	Gel shift, CD, ANS	PEN3	Signaling hub^5^	[56,80,81,82]
CML38	At1g76650	4	?	Gel shift	RALF1, PEN3	Signaling hub^5^	[56,80,83,84]
CML39	At1g76640	4	?	Gel shift	?	Development (Seed, Fruit)	[80,85,86]
CML40	At3g01830	2	?	?	?	?	
CML41	At3g50770	4	?	Gel Shift	?	Plant immunity	[87]
CML42	At4g20780	3	3	CD, ITC, ANS, NMR, HIC, Gel shift	KIC	Signaling hub ^5^	[30,88,89]
CML43	At5g44460	3	3	CD, ITC, ANS, NMR, DSC, HIC, Gel shift	?	Plant Immunity (positive regulation)	[31,88]
CML44	At1g21550	3	?	?	?	?	
CML45	At3g29000	3	?	?	?	?	
CML46	At5g39670	3	?	?	?	Plant Immunity (negative regulation)	[90]
CML47	At3g47480	2	?	?	?	Plant Immunity (negative regulation)	[90]
CML48	At2g27480	2	?	?	?	?	
CML49	At3g10300	2	?	?	?	?	
CML50	At5g04170	2	?	?	?	?	

^1^ Name according to [13]. The name assigned to four accession numbers differs between [13] and UniProt. At3g59450: CML1 [13], CML46 [UniProt]; At2g15680: CML30 [13], CML1 [UniProt]; At3g29000: CML45 [13], CML30 [UniProt]; At5g39670: CML46 [13], CML45 [UniProt]. ^2^ Number of EF-hands based on PROSITE-ProRule prediction [91]. Not all motifs found are actually functional and differences exist between the predicted motifs by MEME (Figure 2) and PROSITE-ProRule. ^3^ Number of functional Ca^2+^-binding sites as experimentally measured by ITC and/or NMR analysis. ^4^ Techniques used to assess structural and Ca^2+^-binding properties. Gel shift to study electrophoretic mobility; ITC to study thermodynamic parameters of metal-binding; ANS and HIC to evaluate surface-exposed hydrophobicity; CD and NMR spectroscopy to evaluate conformational changes in secondary and tertiary structure; DSC and LP to assess thermal and structural stability; MM, molecular modeling. ^5^ Role as key hub in plant development and response to both biotic and abiotic stresses. ?, no information available.

## Abbreviations

CaM	Calmodulin
CML	CaM-like protein
CD	Circular dichroism
ITC	Isothermal titration calorimetry
DSC	Differential scanning calorimetry
HIC	Hydrophobic interaction chromatography
NMR	Nuclear magnetic resonance
Cryo-EM	Cryo-electron microscopy
HSQC	Heteronuclear single-quantum coherence
ANS	Anilino-8-naphthalene sulfonate
SEC	Size-exclusion chromatography
LP	Limited proteolysis
MM	Molecular modeling
CaMBD	CaM-binding domain
*K*_d_	Dissociation constant
